# Comparison of the success rate after debridement, antibiotics and implant retention (DAIR) for periprosthetic joint infection among patients with or without a sinus tract

**DOI:** 10.1186/s12891-021-04756-x

**Published:** 2021-10-21

**Authors:** Wang Deng, Rui Li, Hongyi Shao, Baozhan Yu, Jiying Chen, Yixin Zhou

**Affiliations:** 1 Department of Orthopedic Surgery, Beijing Jishuitan Hospital, Fourth Clinical College of Peking University, No.31 Xinjiekou East Road, Xicheng District, Beijing, 100035 China; 2 Department of Orthopaedics, Chinese PLA General Hospital, No. 28 Fuxing Road, Haidian District, Beijing, 100039 China; 3 Department of Orthopaedics, Bao Ding Gem Flower Eastern Hospital, Bao Ding, Hebei China

**Keywords:** Debridement, antibiotics and implant retention (DAIR), Periprosthetic joint infection (PJI), Sinus tract

## Abstract

**Background:**

The relevance between the presence of a sinus tract and the failure risk after debridement, antibiotics and implant retention (DAIR) for periprosthetic joint infection (PJI) after hip or knee arthroplasty is still unclear. This study aimed to compare the success rate of DAIR between patients with or without a sinus tract and to explore the possible risk factors for failure after DAIR in patients with a sinus tract.

**Methods:**

Consecutive DAIR cases for PJI after hip or knee arthroplasty between January 2009 and June 2019 with a minimum 1-year follow-up in two tertiary joint arthroplasty centers were included. Patients were classified into the sinus tract group and the non-sinus tract group according to the presence of a sinus tract. The success rate after DAIR were compared using Kaplan-Meier survival analysis. Potential risk factors for failure in the sinus group were also explored.

**Results:**

One hundred seven patients were included. At a median 4.4 years of follow-up, 19 of 52 (36.5%) cases failed in the sinus tract group, while 15 of 55 (27.3%) cases failed in the non-sinus tract group. The 1-year and 5-year cumulative success rates were 71.2% (95% confidence interval (CI): 59.8-84.6%) and 56.8% (95% CI: 42.6-75.7%) in the sinus tract group, respectively, which were similar to the counterparts in the non-sinus tract group (*P* = 0.214). Among patients with a sinus tract, DAIR with the exchange of modular components showed a higher success rate (75.8% versus 47.4%, *P* = 0.038).

**Conclusions:**

The presence of a sinus tract does not affect the success rate of DAIR. Modular component exchange in DAIR was proposed for patients with a sinus tract for an improved infection control rate.

## Background

Periprosthetic joint infection (PJI) is one of the most devastating complications after joint arthroplasty, with an incidence of 1-2% [1, 2]. The total number of PJI cases will increase due to the surge of new arthroplasties worldwide and previously implanted prostheses [2, 3]. Treatment of PJI brings great suffering to patients, poses difficult challenges to surgeons and causes huge financial burdens [4, 5]. The main treatment options involve debridement, antibiotics and implant retention (DAIR), one-stage and two-stage revision [6].

Compared with one-stage or two-stage revision, DAIR has the advantages of lower cost, lower skill requirement and better postoperative joint functions [7–9]. Numbers of cases treated with DAIR have increased rapidly in recent years [6, 10, 11]. However, the infection control rate after DAIR ranges from 10 to 100% in different studies, and the overall infection control rate in a recent meta-analysis is approximately 60% [12]. Identifying the possible risk factors for failure after DAIR is of great clinical significance since it may help to ensure higher infection control rate [13]. As an important sign of deep infection, a sinus tract could present in either acute or chronic infection [14–16]. However, data regarding the presence of a sinus tract were not reported in most studies that aimed to explore the risk factors for failure after DAIR [17–20]. Recent meta-analyses also did not mention this topic [12, 21, 22]. Patients with a sinus tract do go through DAIR to treat PJI in the clinic, and the relevance between the presence of a sinus tract and the failure risk after DAIR is still unclear [23–27]. To our knowledge, no studies have directly focused on the comparison of the infection control rate after DAIR for PJI after hip or knee arthroplasty between patients with or without a sinus tract, nor have any studies explored the possible risk factors for failure after DAIR in patients with a sinus tract.

The aims of this study were to (1) compare the PJI control rate after DAIR between patients with or without sinus tract and (2) explore the possible risk factors for failure after DAIR in patients with a sinus tract in a multicenter study.

## Methods

### Patient inclusion

Following Institutional Review Board approval, this study was performed with consecutive hip or knee PJI cases treated with DAIR from January 2009 to June 2019 in two tertiary joint arthroplasty centers. The inclusion criteria also involved (1) meeting the 2013 International Consensus Meeting (ICM) definition of PJI after hip or knee arthroplasty [28]; (2) DAIR under open arthrotomy; (3) first time surgical treatment for PJI and (4) at least one year of follow-up. The exclusion criteria were as follows: (1) superficial site infection and (2) index surgery using mega-prosthesis. Data on demography, joint surgical history and infection classifications were retrieved from the medical records. Acute infection was defined when the interval time from index surgery to DAIR was less than 4 weeks. If an abrupt onset of infection symptoms (e.g., fever, swelling, tenderness, wound drainage, etc.) occurred in a prior symptom-free prosthetic joint 4 weeks after the index surgery and the symptom duration from onset to DAIR was less than 3 weeks, the cases were defined as acute hematogenous PJI. Otherwise, the infection was considered chronic [29, 30]. The sinus tract was defined and verified intraoperatively as a channel communicating the outside and the joint cavity [14]. All patients were divided into the sinus tract group and the non-sinus tract group according to the presence of a sinus tract.

### Treatment protocol

All the included cases underwent DAIR by fellowship-trained arthroplasty surgeons in two tertiary joint arthroplasty centers in our region. The two centers shared the same therapeutic protocol. With the definite or suspected diagnosis of PJI, a multidisciplinary team made the decision in favor of DAIR with the consent of the patient after fully understanding of all the treatment options, including one-stage or two-stage revision, DAIR or antibiotic suppressive treatments and their possible risks and benefits. A posterior lateral approach for hip and midline incision with a medial patellar approach for the knee was used for open arthrotomy. Sinus tracts were explored and excised roundly after confirmation of the communication with the joint cavity. Implants were assessed as well-fixed for DAIR; otherwise, one-stage or two-stage revisions were performed. After taking the samples for microbial cultivation and joint fluid analysis, radical synovectomy around the joints and excision of all the potentially infected tissue and sequestrum were performed. After thorough pulse lavage with antiseptic saline, 3% hydrogen peroxide and diluted iodophor solutions were used as anti-infection agents for five minutes and were repeated at least twice during surgery. Modular components were removed for thorough debridement. Exchange to new modular components or reinsertion of the original ones was decided by the surgeons after considering the reusability of original ones and the availability of new ones. Utilization of topical antibiotics was also decided by the surgeons. Commonly, two grams vancomycin were chosen as the topical antibiotics for PJI caused by gram-positive bacteria or unknown pathogen while one gram meropenem was used for PJI with gram-negative bacteria.

If the microbial culture results were available before the debridement, antibiotics were chosen under the guidance of antibiotic susceptibility tests and advice from pharmacists. Vancomycin and a third-generation cephalosporin were combined to cover both gram-positive and gram-negative bacteria for patients without preoperative microbial culture results. Organism-specific antibiotics were administered when the intraoperative culture results were reported. For culture-negative cases, we maintained these broad-spectrum antibiotics [31]. Patients received intravenous antibiotics for at least two weeks and then oral antibiotics for at least another four weeks. During the hospitalization, clinical symptoms, such as swelling, redness, wound drainage, were checked when changing the wound dress. Infection biomarkers, including blood routine test, CRP and ESR, were tested every other day in the first week and once a week afterwards. If the clinical symptoms subsided and the infection biomarker decreased, we continued the wound care and antibiotic administration in accordance with the protocol. If the symptoms did not relive or the biomarker was still high or raised again after a drop, subsequent surgical intervention or continuous suppressive antibiotic therapy was decided by the multidisciplinary team and the patients. Postoperative functional recovery after DAIR began after removing the drainage tube. The discharging criteria included the relief of infection symptoms, the decrease and stabilization of infection biomarkers. The total duration of antibiotics was between 6 to 12 weeks [32].

### Follow-up and outcome assessment

After being discharged from the hospitals, we encouraged patients to attend clinic visits routinely and to return to the hospital as soon as possible if any infection symptoms occurred. The success of DAIR was defined according to the definition suggested by Diaz-Ledezma et al.: (1) infection eradication, characterized by a healed wound without sinus tracts, drainage, or pain, and no infection recurrence caused by the same organism strain; (2) no subsequent surgical intervention for infection after DAIR; and (3) no occurrence of PJI-related mortality (by causes such as sepsis and necrotizing fasciitis) [33]. Cases with chronic infection symptoms and treated with antibiotic suppressive therapy were defined as failure. If the DAIR was defined as failed, we recorded the time of failure, subsequent treatment options and the status of the joint at the last follow-up.

### Statistical analysis

In the comparison between the sinus tract and non-sinus tract groups, continuous variables in demographic data were expressed as the mean with range or the median with interquartile range (IQR) and were compared by Student’s T-test or Mann-Whitney U test according to the Kolmogorov-Smirnov test of normality. Categorical variables were compared between two groups using Chi-square test or Fisher’s exact test. The DAIR outcomes were depicted and compared between the two groups using a Kaplan-Meier survival analysis (log-rank test), and the cumulative 1-year and 5-year infection control rates were reported. Among patients with a sinus tract, possible risk factors were also compared between the successful and failed groups. Kaplan-Meier survival analysis was also performed for the potential risk factors. The significance level was set at a *P*-value < 0.05.

## Results

One hundred eighteen patients were reviewed initially, and 107 cases were included after excluding 9 patients for superficial site infection and 2 for mega-prosthesis. Fifty-two patients showed the presence of a sinus tract, and the other 55 patients were classified into the non-sinus tract group. The preoperative data, including basic demography, joint history (hip or knee, primary or revision) and infection types, were comparable between the two groups. The pathogen classification, rate of modular component exchanges and topical antibiotic administration were also similar between the two groups (Table 1).

**Table 1:** The comparison of preoperative and intraoperative data between the sinus tract group and non-sinus tract group

Variables	Sinus tract (*n* = 52)	Non-sinus tract (*n* = 55)	P-Value
Age, yrs. (mean ± SD)	59.46 ± 16.93	63.48 ± 11.64	0.085^a^
Male, n (%)	26 (50.0%)	19 (34.5%)	0.106^b^
BMI, kg/m^2^ (mean ± SD)	26.29 ± 5.63	26.82 ± 4.17	0.581^a^
Median CCI, (IQR)	3 (2, 4)	3 (2, 4)	0.317^c^
Joint (Hip), n (%)	17 (32.7%)	12 (21.8%)	0.206^b^
Revision, n (%)	12 (23.1)	8(14.5)	0.258^b^
PJI type, n (%)			0.182^b^
Early PJI	11 (21.2%)	14 (25.5%)	
Acute hematogenous PJI	16 (30.8%)	24 (43.6%)	
Chronic PJI	25 (48.1%)	17 (30.9%)	
Culture positive, n (%)	36 (69.2%)	36 (65.5%)	0.677^b^
Organism			0.265^d^
Staphylococcus (MR)	12 (21.3%)	10 (18.2%)	
Staphylococcus (MS)	3 (5.8%)	5 (9.1%)	
Gram-negative	6 (11.5%)	5 (9.1%)	
Other pathogens	6 (11.5%)	13 (23.6%)	
Polymicrobial	9 (17.3%)	3 (5.5%)	
Culture negative	16 (30.8%)	19 (34.5%)	
Modular exchange, n (%)	34 (65.4%)	43 (78.2%)	0.141^b^
Topic antibiotics, n (%)	9 (17.3%)	12 (21.8%)	0.557^b^
Median follow-up, yrs. (IQR)	3.18 (1.80, 7.68)	5.38 (2.42, 7.98)	0.182^c^

*SD* Standard deviation, *CCI* Charlson Comorbidity Index, *IQR* Interquartile range, *PJI* Periprosthetic joint infection, *MR* methicillin-resistant, *MS* methicillin-sensitive; ^a^ Student’s T-test; ^b^ Chi-squared test; ^c^ Mann–Whitney U test; ^d^ Fisher’s exact test

**Fig. 1:**
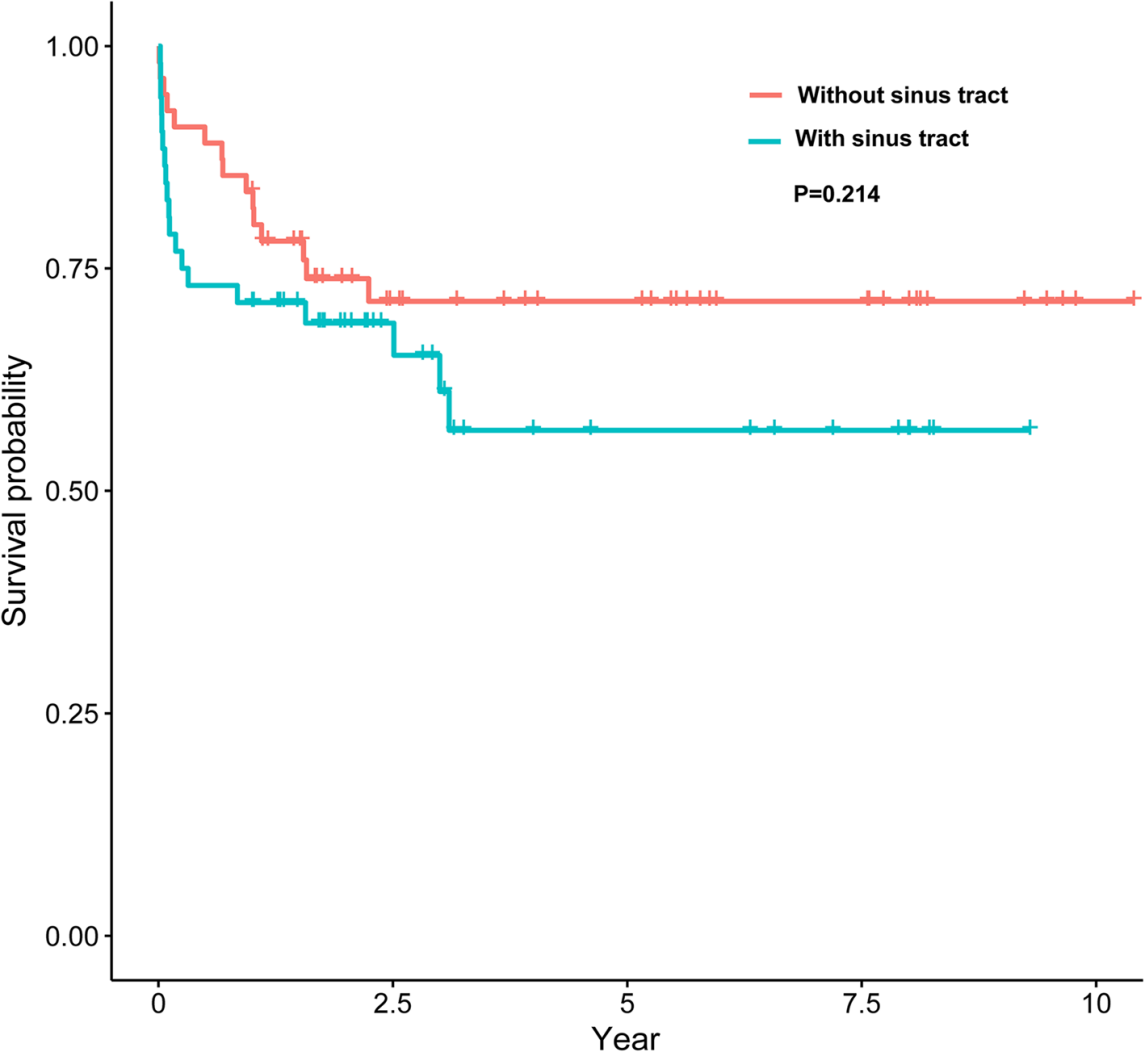
The Kaplan-Meier survival curves for DAIR comparing the sinus tract group and non-sinus tract group

The median follow-up time in all the patients was 4.4 (IQR: 2.05-7.91) years, and there was no significant difference between the two groups in follow-up time. At the last follow-up, 19 cases (36.5%) had failed, and 3 cases (5.8%) had died unrelated to PJI in the sinus tract group. Fifteen cases (27.3%) had failed in the non-sinus tract group. The further treatment after the failed DAIR included suppressive antibiotic therapy, repeated DAIR, one-stage and two-stage exchange revision (Table 2). The cumulative infection control rate curve was depicted by the failure of DAIR as an end-point event, and the two groups showed similar cumulative infection control survivorship in the Kaplan-Meier curve (Fig. 1, *P* = 0.214). The 1-year and 5-year cumulative infection control rates were 71.2% (95% confidence interval (CI): 59.8-84.6%) and 56.8% (95% CI: 42.6-75.7%) in the sinus tract group, respectively, while those in the non-sinus tract group were 83.6% (95% CI: 74.4-94.0%) and 71.3% (95% CI: 59.9-84.9%), respectively.

**Table 2:** Further treatment after the failed debridement, antibiotics and implant retention (DAIR) in the sinus tract group and non-sinus tract group

Further treatment^a^	Sinus tract		Non-sinus tract	
	Total (*n* = 19)	Success^b^ 7 (36.8%)	Total (*n* = 15)	Success^b^ 10 (66.7%)
Suppressive antibiotic therapy	5 (26.3%)	–	3 (20.0%)	–
Multiple DAIR	6 (31.6%)	3 (50.0%)	1 (6.7%)	1(100.0%)
One-stage exchange revision	3 (15.8%)	2 (66.7%)	0 (0.0%)	–
Two-stage exchange revision	5 (26.3%)	2 (40.0%) ^c^	11 (73.3%)	9 (81.8%)

^a^Data are presented as numbers of patients (percentages); ^b^ The percentages indicate the proportion of patients who have infection control after the corresponding treatments; ^c^ Another two patients have finished successful spacer implantation and are waiting for the two-stage revision

**Fig. 2:**
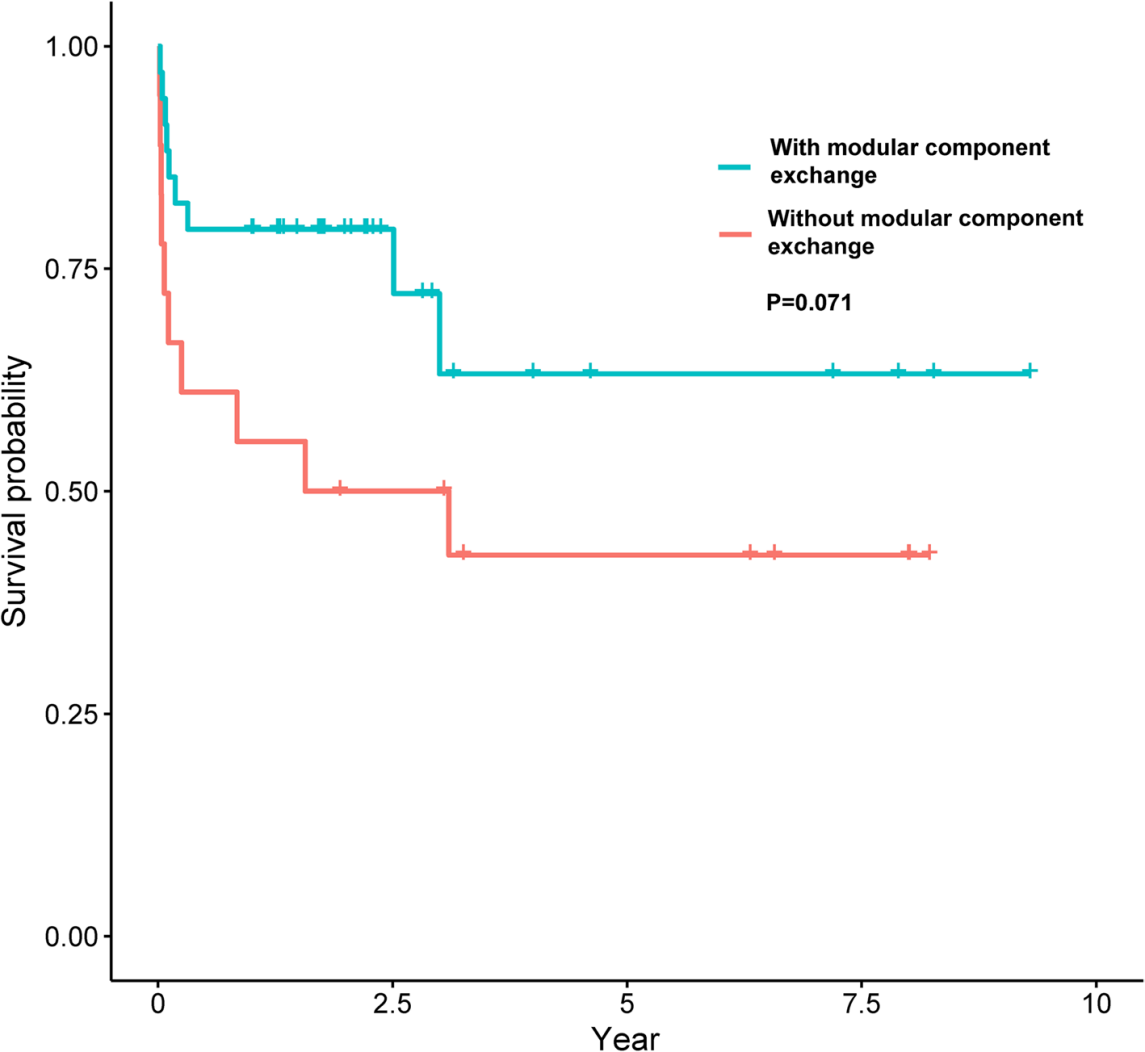
The Kaplan-Meier survival curves for DAIR with or without exchange of modular component in the sinus tract group

**Fig. 3:**
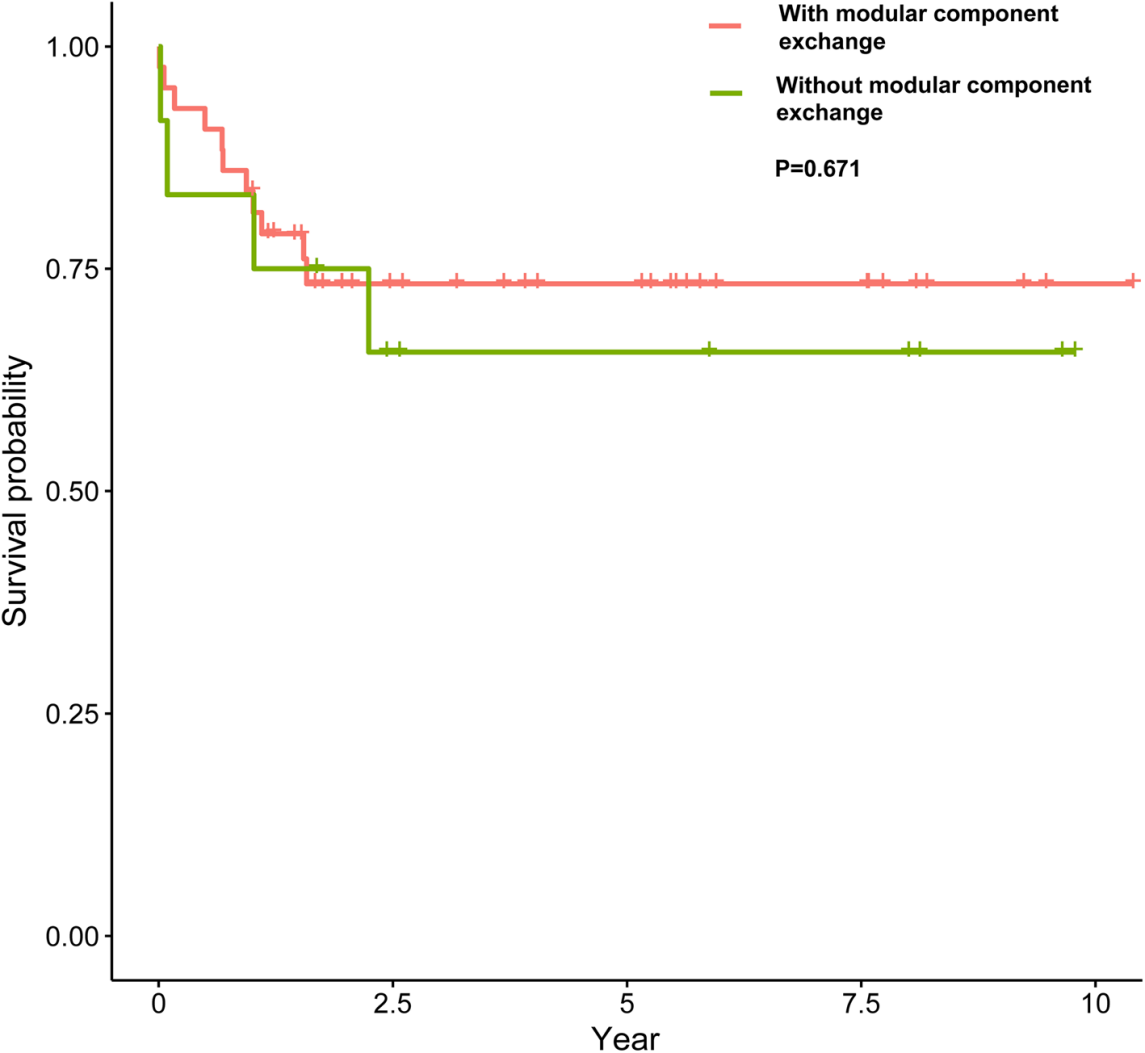
The Kaplan-Meier survival curves for DAIR with or without exchange of modular component in the non-sinus tract group

Among the patients with a sinus tract, the successful group showed a higher proportion of modular component exchanges than the failed group (75.8% vs. 47.4%, *P* = 0.038) (Table 3). Patients after modular component exchange showed better infection control survivorship than patients without modular component exchange at the very edge of significance (Fig. 2, *P* = 0.071). Furtherly, we depicted the Kaplan-Meier curve among the patients without a sinus tract and the exchange of modular components did not influence the infection control survivorship among patients without a sinus tract (Fig. 3, *P* = 0.671).

**Table 3:** The comparison of preoperative and intraoperative data between the successful and failed patients in the sinus tract group

Variables	Success (*n* = 33)	Failure (n = 19)	P-Value
Age, yrs. (mean ± SD)	62.76 ± 14.03	53.74 ± 20.19	0.096^a^
Male, n (%)	15 (45.5%)	11 (57.9%)	0.746^b^
BMI, kg/m^2^ (mean ± SD)	25.43 ± 5.78	27.79 ± 5.17	0.148^a^
Median CCI, (IQR)	3 (2,4)	2 (1,4)	0.224^c^
Joint (Hip), n (%)	9 (27.3%)	8 (42.1%)	0.272^b^
Revision, n (%)	7 (21.2%)	5 (26.3%)	0.937^d^
PJI type, n (%)			0.490^b^
Early PJI	6 (18.2%)	5 (26.3%)	
Acute hematogenous PJI	12 (36.4%)	4 (21.1%)	
Chronic PJI	15 (45.5%)	10 (52.6%)	
Culture positive, n (%)	24 (72.7%)	12 (63.2%)	0.472^b^
Modular exchange, n (%)	25 (75.8%)	9 (47.4%)	**0.038**^b^
Topic antibiotics, n (%)	4 (12.1%)	5 (26.3%)	0.356^d^

SD: Standard deviation; CCI: Charlson Comorbidity Index; IQR: Interquartile range; PJI: Periprosthetic joint infection; ^a^ Student’s T-test; ^b^ Chi-squared test; ^c^ Mann–Whitney U test; ^d^ Continuity correction

## Discussion

As one of the major treatment options for PJI, the main concerns for DAIR is the unstable infection control rate in different studies [12]. Proper patient selection may help to ensure infection control rate after DAIR [13]. In this study, we first reported the 1-year and 5-year cumulative infection control rates (70.6 and 56.3%, respectively) after DAIR for PJI in patients with a sinus tract and found that they were similar to their counterparts in patients without a sinus tract. More importantly, modular component exchange could improve the infection control rate in patients with a sinus tract.

Compared with one-stage and two-stage revision, DAIR avoids the significant challenges in removing the fixed prostheses, making joint reconstruction and increasing the risk of perioperative and postoperative complications [34]. The other advantages of DAIR include lower cost and better postoperative joint functions. As estimated, the cost in a two-stage revision is 2-3 times that in a DAIR case [9, 35]. In the postoperative comparison between different PJI treatment options and non-infected primary arthroplasty, patients after successful DAIR share similar postoperative SF-12, Harris hip score and Western Ontario and McMaster Universities Osteoarthritis Index with patients after non-infected primary joint arthroplasty, while two-stage revision could not achieve comparable joint function scores [7, 8]. These advantages of DAIR are appealing for most orthopedists and patients. Data from the Norwegian Arthroplasty Register showed that the annual number of DAIR cases for PJI after total knee arthroplasty and total hip arthroplasty has exceeded the total number of one-stage and two-stage revisions in recent years [6, 10]. Expansion of DAIR in the treatment of PJI is in progress [25], and exploration of the possible risk factors for failure is of great importance.

The presence of a sinus tract was once recognized as a risk factor for failure after DAIR [28]. However, among all the previous publications that explored the potential risk factors for failure after DAIR, only the study from Marculescu et al. showed that sinus tracts could statistically increase the failure risk (Table 4) [15, 23–27, 36, 37]. Five studies did not reach a significant difference [15, 25–27, 36], while two studies reported a lower failure rate in patients with a sinus tract [23, 24]. Whether the presence of a sinus tract should be regarded as a risk factor is still a matter of debate. One of the possible reasons why only the study from Marculescu et al. reached a significant difference is the time of DAIR treatment. The cases reported by Marculescu et al. were performed during 1995-1999, while most DAIR cases included in the other seven studies were performed after 2000 [37]. In a recent meta-analysis, Kunutsor et al. reported that the overall infection control rate of DAIR performed before 2000 was only 51.50%, which was lower than that of DAIR performed after 2000 (61.2%, *P* = 0.028) [12]. This improvement may be due to the amelioration of surgical techniques and the optimization of antibiotic administration [12, 18]. The increase in the infection control rate after DAIR with time may have resulted in a better chance for infection control in patients with a sinus tract. In our study, all DAIRs were performed from 2009 to 2019, and the overall infection control rate was 67.3%, which was slightly better than the data provided in Kunutsor et al.’s meta-analysis (61.2%) [12]. Over 70% of patients with a sinus tract could achieve infection control at the 1-year follow-up and over half at the 5-year follow-up. Anti-infection agents were recommended in debridement even without specific protocols available [13]. In our debridement process, we used repeated anti-infection agents (3% hydrogen peroxide and diluted iodophor solutions) in surgery according to the experience from traumatology, which may also contribute to the acceptable infection control rate in patients with a sinus tract.

**Table 4:** The comparison of success rate between the sinus tract group and non-sinus tract group in previous studies

Study	Centers/Nations	DAIR time	Sinus tract N (%)		Non-sinus tract N (%)		HR or OR (95%CI)	P-value
			Success	Failure	Success	Failure		
Mu et al. 2020 [15]	Single center from China	2011-2018	30^a^		43^a^		0.319 (0.062-1.631)^c^	0.170
Shohat et al. 2020 [23]	27 centers throughout the USA and Europe	2005-2017	212 (70.2)	90 (29.8)	557(63.9)	315 (36.1)	–	0.049^d^
Lesens et al. 2018 [25]	6 centers from France	2010-2014	39^a^		98^a^		1.8 (0.92-3.81)^b^	0.079
Lowik et al. 2018 [24]	3 centers from Netherlands	2006-2016	32 (78.0)	9 (22.0)	206 (59.7)	139 (40.3)	0.42(0.19-0.90)^c^	0.022
Lora-Tamayo et al. 2017 [26]	52 centers throughout 15 nations	2003-2012	34 (55.7)	27 (44.3)	223 (59.0)	155 (41.0)	1.12(0.75-1.69)^b^	0.58
Tornero et al. 2015 [27]	Database from Spain	1999-2014	12 (70.6)	5 (29.4)	73 (76.0)	23 (24.0)	1.32(0.42-4.15)^c^	0.761
Betz et al. 2015 [36]	Single center from Switzerland	1996-2012	2 (100)	0 (0)	29 (80.6)	7 (19.4)	0	1.000
Marculescu et al. 2006 [37]	Single center from USA	1995-1999	15^a^		84^a^		2.85(1.50-5.44)^b^	0.002

^a^Exact failure number in each group was not available in the corresponding literature; ^b^HR; ^c^OR; ^d^Chi-squared test

The presence of a sinus tract has been pathognomonic for deep infection throughout surgical history and is recommended as one of the major criteria for the diagnosis of PJI [14, 38]. Although the surgical management of sinus tracts has been widely discussed, few studies have provided a detailed pathologic description of sinus tracts in PJI. A sinus tract could present in either acute or chronic infection, or evolve from prolonged postoperative drainage [14–16]. A recent prospective observational cohort study of 783 PJI patients from 27 centers in Australia and New Zealand reported that sinus tracts existed among 46 (23.4%) of the 196 early PJIs [16]. Mu et al. also reported 73 patients treated with DAIR and topical antibiotics delivery for PJI within 3 months after the primary joint arthroplasty and the presence of sinus tract was high to 41% [15]. Even though this proportion is lower than that in chronic PJI (47.9%), it still suggests that a sinus tract do not necessarily indicate chronic infection. Xu et al. reported five risk factors for the presence of a sinus tract, which included smoking (OR = 1.83), hypothyroidism (OR = 1.62), hypoalbuminemia (OR = 1.52), hip joint involvement (OR = 1.43), and prior revision surgery (OR = 1.37) [39]. To our knowledge, no previous study reported specific risk factors for failure after DAIR in patients with a sinus tract.

In the exploration of possible risk factors for failure after DAIR among patients with a sinus tract, we noticed that modular component exchange could improve the PJI control rate at the last follow-up (75.8% vs. 47.4%, *P* = 0.038) and showed better infection-free survivorship in Kaplan-Meier survival analysis with a strong tendency towards statistical significance (*P* = 0.071). Modular component exchange was performed in nearly 72% of DAIR cases in the current study, which was similar to the national survey results that indicated that 74 of 99 institutions (75%) performed modular component exchange for hip PJI and 82% for knee PJI in the Netherlands [40]. Modular component exchange has been recommended in ICM consensus after reviewing the present literature and available evidence [13, 28]. In the failure predictive score reported by Wouthuyzen-Bakker et al., modular component exchange is also included as a protective factor [41]. Removing the modular components allows for better visualization and access of the articular cavity, especially in the posterior or deep aspect, and a larger working space, thereby facilitating more thorough debridement. At the same time, the exchange of modular components could directly reduce the bioburden and further help to eliminate the PJI. However, the exchange of modular components may be impeded by the lack of component supply in clinical practice and can increase the cost. DAIR without modular component exchange in patients with a sinus tract should be avoided.

This study has several limitations. First, as a retrospective cohort study, some inherent constraints may be embedded. However, it is impractical to perform studies with higher evidence to compare the infection control rates in patients with or without sinus due to the low incidence and urgent patient conditions. Second, the limitation of patient sample size may impede the detection of a significant difference in the comparison of cumulative infection-control survivorship between the two groups. However, useful information about the cumulative infection control rate in each group could also be provided by the Kaplan-Meier curve. Third, the pathogenic microorganisms were different between patients, as were the debridement skills among surgeons. Antibiotic administration may not be consistent in each patient. However, all these patients shared the same therapeutic protocol. Fourth, the follow-up time for some of the patients has not yet reached 5 years, the middle-term follow-up time set by the consensus meeting, meaning that some patients may experience failure in the future [42]. Longer follow-ups should be performed, and more patients should be included in future studies. Finally, since this study only focused patients after DAIR and did not compare them with patients after one-stage or two-stage revision, more future research still needs to be performed for the exploration of the best treatment options for PJI.

## Conclusions

The presence of a sinus tract does not affect the success rate of DAIR. Modular component exchange in DAIR was proposed for patients with a sinus tract for an improved infection control rate.

## Data Availability

The data that support the findings of this study are available from the corresponding authors but restrictions apply to the availability of these data, which were used under license for the current study, and so are not publicly available. Data are however available from the authors upon reasonable request and with permission of the hospital ethical institutional review board.

## Abbreviations

DAIR: Debridement, antibiotics and implant retention (DAIR)
PJI: Periprosthetic joint infection
ICM: International Consensus Meeting
